# An Investigation of Extended-Dimension Embedded CKF-SLAM Based on the Akaike Information Criterion

**DOI:** 10.3390/s24237800

**Published:** 2024-12-05

**Authors:** Hanghang Xu, Yijin Chen, Wenhui Song, Lianchao Wang

**Affiliations:** College of Geoscience and Surveying Engineering, China University of Mining and Technology (Beijing), Beijing 100083, China; bqt2000205068@student.cumtb.edu.cn (H.X.); songwh0820@163.com (W.S.); bqt1900205063@student.cumtb.edu.cn (L.W.)

**Keywords:** truncated singular-value decomposition, Akaike information criterion, dimensionality expansion processing, embedded CKF, simultaneous localization and map construction

## Abstract

Simultaneous localization and mapping (SLAM) faces significant challenges due to high computational costs, low accuracy, and instability, which are particularly problematic because SLAM systems often operate in real-time environments where timely and precise state estimation is crucial. High computational costs can lead to delays, low accuracy can result in incorrect mapping and localization, and instability can make the entire system unreliable, especially in dynamic or complex environments. As the state-space dimension increases, the filtering error of the standard cubature Kalman filter (CKF) grows, leading to difficulties in multiplicative noise propagation and instability in state estimation results. To address these issues, this paper proposes an extended-dimensional embedded CKF based on truncated singular-value decomposition (TSVD-AECKF). Firstly, singular-value decomposition (SVD) is employed instead of the Cholesky decomposition in the standard CKF to mitigate the non-positive definiteness of the state covariance matrix. Considering the effect of small singular values on the stability of state estimation, a method is provided to truncate singular values by determining the truncation threshold using the Akaike information criterion (AIC). Furthermore, the system noise is embedded into the state variables, and an embedding volume criterion is used to improve the conventional CKF while extending the dimensionality. Finally, the proposed algorithm was validated and analyzed through both simulations and real-world experiments. The results indicate that the proposed method effectively mitigates the increase in localization error as the state-space dimension grows, enhancing time efficiency by 55.54%, and improving accuracy by 35.13% compared to the standard CKF algorithm, thereby enhancing the robustness and stability of mapping.

## 1. Introduction

Data filtering has a wide range of applications in signal processing, target tracking, and satellite navigation [1]. The Kalman filter (KF) is a commonly used solution for state estimation in linear systems [2,3]. However, the kinematic equations are nonlinear in the pose estimation of simultaneous localization and mapping (SLAM), where the accurate estimation of the robot’s pose is crucial for concurrent environmental mapping. Accurate and robust pose estimation is crucial to the reliability of the localization process, which plays a vital role in SLAM. Currently, SLAM mainly uses KF, extended Kalman filter (EKF), or Particle Filter (PF) as its core algorithms [4]. For state estimation in nonlinear systems, researchers have proposed the EKF [5,6,7], unscented Kalman filter (UKF) [8,9], CKF [10], along with several improved algorithms [11,12]. The EKF uses a first-order Taylor series expansion to linearize the nonlinear equations of the system. The UKF applies a sampling approximation method through the Unscented Transform (UT) [13] to estimate the state of nonlinear systems [14,15]. The CKF selects cubature points in the state space to propagate and update the state, without relying on linearization assumptions. Reference [16] pointed out that CKF faces challenges such as low real-time performance, large computational complexity, and low tracking accuracy when dealing with dynamic targets. To address these challenges associated with CKF, researchers have proposed several improvements. Reference [17] utilized the embedded cubature criterion to calculate probability integrals in nonlinear approximations, achieving higher precision filtering estimation [18]. Reference [19] employed an inverse Wishart distribution to model the covariance matrix of prediction error and measurement noise, addressing the issues of unknown state noise and uncertain measurement noise. Reference [20] used the Huber method to update the square root CKF algorithm in the form of nonlinear statistical regression. When system observations are disturbed by abnormal noise, the affected data were corrected by truncating the observations, which improved the problem of noise distribution deviating from the Gaussian assumption due to abnormal interference. However, these methods do not address the problem that, as the filtering error of the system increases with the dimensionality of the state space, system noise information cannot propagate through nonlinear functions, resulting in outliers in the system-state-driven model and observation data. This leads to instability in the system model.

Motivated by these limitations, this paper proposes a truncated singular-value decomposition extended-dimensional embedded CKF (TSVD-AECKF) algorithm for pose estimation in mobile-robot SLAM. Firstly, singular-value decomposition (SVD) is employed in place of the Cholesky decomposition used in the standard CKF. Due to the sensitivity of system stability to minor variations in singular values, this paper introduces a truncation process and presents a method for determining the truncation threshold. Secondly, the system noise is incorporated into the state variables, thereby expanding the dimensionality of the system. The embedded CKF (E-CKF) algorithm is employed to mitigate positioning errors that grow with the increasing state dimensionality. Finally, multiple simulations were conducted under diverse conditions. The results show that the proposed method enhances the robustness of SVD against system noise interference and addresses the challenge of multiplicative noise propagation, achieving accurate positioning in high-dimensional systems.

The rest of this paper is organized as follows. Section 2 provides an overview of the SLAM model, followed by Section 3 and Section 4, which introduce the detailed solution algorithm. Before concluding, Section 4 presents comparison simulations along with an analysis of the simulation data.

(1) After conventional singular-value decomposition, smaller singular values can lead to larger errors, causing solution instability. This paper proposes and uses the Akaike information criterion (AIC) to determine the truncation threshold for singular values.
(2) Considering the inherent flaws of non-additive noise in the system and the spherical radial volume criterion in the standard CKF algorithm, an embedded approach is applied to the extended-dimensional CKF algorithm.
(3) An AIC-based extended embedded CKF algorithm is proposed, with detailed time and measurement update equations provided.

## 2. SLAM

### 2.1. Description of the SLAM

**Figure 1:**
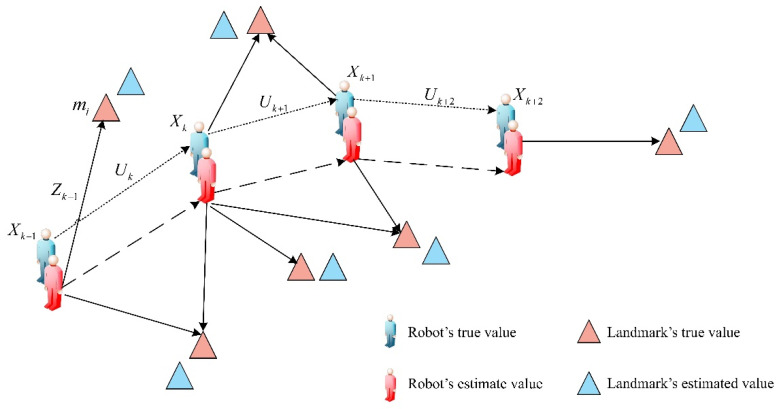
SLAM model.

SLAM refers to the process by which mobile robots use their own sensors to simultaneously localize themselves and incrementally build a map in an unknown environment. The core of SLAM is the probabilistic estimation of the system state throughout the entire trajectory. Consider a mobile robot navigating in an unknown environment, using its sensors to observe previously unknown landmarks, as illustrated in Figure 1 [21], where $x_{k}$ is the pose-state vector of the mobile robot, $u_{k}$ is the control vector that drives the mobile-robot state from time k−1 to time k, $m_{i}$ is the position-state vector of the ith static environment feature, and $z_{i,k}$ is the vector with the i-th static environmental feature observed by the mobile robot at time k. In the SLAM system, the process mainly comprises three sequential components: state prediction, landmark observation, and state update, which are iteratively repeated. Specifically, the robot’s current pose is initially predicted based on its previous pose and the motion model. Next, landmark observations are gathered using sensors like LiDAR or cameras, and these observations are associated with the robot’s pose. Finally, both the robot’s pose and landmark information are updated using the obtained observations. Through the continuous iteration of these three components, the robot can localize itself and incrementally construct a map of the environment, thereby achieving autonomous localization and navigation.

### 2.2. SLAM Probability Model

The theory of Bayesian filtering serves as the foundational framework for studying SLAM algorithms [22]. In SLAM, the process of robot localization and map construction is commonly framed as a state estimation problem. Essentially, the SLAM problem involves calculating the joint posterior probability density function of the robot’s pose $x_{k}$ and the positions m of environmental landmarks at the current time step, based on the observation information $Z_{0,k}$ and control inputs $U_{0,k}$ up to time step k [23]:

(1) $$P\left(\left.x_{k},m\right|Z_{0,k},U_{0,k},x_{0}\right)$$

Here, $X_{0,k}=\left\{x_{0},x_{1},\ldots ,x_{k}\right\}=\left\{X_{0,k-1},x_{k}\right\}$ is the historical information of the pose state of the robot, $U_{0,k}=\left\{u_{0},u_{1},\ldots ,u_{k}\right\}=\left\{U_{0,k-1},u_{k}\right\}$ is the historical information of the control input, $Z_{0,k}=\left\{z_{0},z_{1},\ldots ,z_{k}\right\}=\left\{Z_{0,k-1},z_{k}\right\}$ is the set of all observations, and $m=\left\{m_{1},m_{2},\ldots ,m_{n}\right\}$ is the set of all landmarks. The joint-state probability distribution at time k can be calculated using the Bayesian theorem, considering the joint-state probability distribution at time k−1 of $P\left(\left.x_{k-1},m\right|Z_{0,k-1},U_{0,k-1}\right)$, the observation vector $z_{k}$, and the control input vector $u_{k}$ at time k.

## 3. CKF

### 3.1. CKF Based on Singular-Value Decomposition

The SVD-CKF algorithm replaces the Cholesky decomposition used in the standard CKF algorithm with SVD when performing an iterative decomposition of the error covariance matrix. This substitution addresses the potential issue of non-positive definiteness in the covariance matrix caused by Cholesky decomposition, thereby enhancing the robustness of the CKF algorithm. However, the use of SVD also introduces new challenges. To investigate the inherent issues in the SVD-CKF algorithm, the computational steps of the SVD-CKF algorithm are presented first. Consider the following discrete-time nonlinear dynamic system:

(2) $$x_{k}=f\left(x_{k-1}\right)+\omega_{k-1},$$

(3) $$z_{k}=h\left(x_{k}\right)+\upsilon_{k}.$$

where $x_{k}$ and $z_{k}$ are the system-state vector and measurement at time k; $f\left(\cdot \right)$ and $h\left(\cdot \right)$ are the nonlinear functions of the system-state transition model and measurement model, respectively; $\omega_{k}$ and $\upsilon_{k}$ are the process noise and observation noise, whose covariance matrices are $Q_{k}$ and $R_{k}$, respectively. The steps of the cubature Kalman filter algorithm based on singular-value decomposition are as follows:

(1) Calculate the cubature sampling points and their corresponding weights [24]. The basic cubature points and their corresponding weights are determined using the third-order cubature rule, as illustrated below [25,26]:

(4) $$\xi_{i}=\sqrt{\frac{N}{2}}{\left[1\right]}_{i},$$

(5) $$\omega_{i}=\frac{1}{N}$$

where i=1,2,…,N, N represents the number of cubature sampling points. Under the third-order cubature rule, the total number of sampling points is twice the state dimension, i.e., N=2n. ${\left[1\right]}_{i}$ denotes the i-th point set $\left[1\right]$, where $\left[1\right]$ refers to the fully symmetric point set. This set is generated by permuting all elements of the unit vector $e={\left[1,0,\ldots ,0\right]}^{T}$ in n-dimensional space and altering the signs of its elements.

(2) Update the time. Taking the square root of the covariance matrix $P_{k-1}$ through SVD:

(6) $$P_{k-1}=U\left[\begin{array}{cc} S & 0\\ 0 & 0 \end{array}\right]V^{T}$$

where S is a diagonal matrix, $S=diag\left\{s_{1},s_{2},\ldots ,s_{n}\right\}$. The SVD of the covariance matrix is as follows:

(7) $$P_{k-1}=U_{k-1}S_{k-1}V_{k-1}^{T},$$

(8) $$X_{i,k-1}=U_{i,k-1}\sqrt{s_{i}}\xi_{i}+{\hat{x}}_{k-1}$$

Calculate the cubature points propagated through the nonlinear state equation:

(9) $$X_{i,k}^{*}=f\left(X_{i,k-1}\right)$$

Calculate the predicted state and predicted covariance:

(10) $$\left\{\begin{array}{l} x_{k/k-1}=\sum_{i=1}^{2n}\omega_{i}X_{i,k}^{*}\\ P_{k/k-1}=\sum_{i=1}^{2n}\omega_{i}X_{i,k}^{*}{X_{i,k}^{*}}^{T}-{\bar{x}}_{k}{\bar{x}}_{k}^{T}+Q_{k-1} \end{array}\right.$$

(3) Update the measurement. Factorize the term:

(11) $$P_{k-1}=U_{k/k-1}S_{k/k-1}V_{k/k-1}^{T}$$

Calculate the cubature points:

(12) $$X_{i,k/k-1}=U_{i,k/k-1}\sqrt{s_{i}}\xi_{i}+x_{k/k-1}$$

Propagate the cubature points through the nonlinear measurement equation:

(13) $$Z_{i,k}=h\left(X_{i,k/k-1}\right)$$

Calculate the predicted measurement $\bar{z}$, innovation covariance $P_{zz,k}$, and cross-covariance estimates $P_{xz,k}$:

(14) $$\left\{\begin{array}{l} {\bar{z}}_{k}=\sum_{i=1}^{2n}\omega_{i}Z_{i,k}\\ P_{zz,k}=\sum_{i=1}^{2n}\omega_{i}Z_{i,k}Z_{i,k}^{T}-{\bar{z}}_{k}{\bar{z}}_{k}^{T}+R_{k}\\ P_{xz,k}=\sum_{i=1}^{2n}\omega_{i}X_{i,k}Z_{i,k}^{T}-{\bar{x}}_{k}{\bar{z}}_{k}^{T} \end{array}\right.$$

Calculate the gain matrix $K_{k}$, and update the state ${\hat{x}}_{k}$ and covariance $P_{k}$:

(15) $$K_{k}=P_{xz,k}/P_{zz,k},$$

(16) $${\hat{x}}_{k}={\bar{x}}_{k}+K_{k}\left(z_{k}-{\tilde{z}}_{k}\right),$$

(17) $$P_{k}=P_{k/k-1}-K_{k}P_{zz,k}K_{k}^{T}$$

The above outlines the process of the SVD-based CKF algorithm.

### 3.2. Truncated Singular-Value Method

The singular value is the “essential information” contained in the data [27,28]. In order to investigate the problems in the SVD-CKF algorithm, singular-value decomposition was performed on the coefficient matrix C through Equation (18), resulting in Equations (19) and (20).

(18) $$C=U\Lambda V^{T}$$

(19) $$U=\left[u_{1},u_{2},\ldots \right],V=\left[v_{1},v_{2},\ldots \right]$$

(20) $$\Lambda =\left[\begin{array}{cc} Z & 0\\ 0 & 0 \end{array}\right],Z=diag\left(\sigma_{1},\sigma_{2},\ldots ,\sigma_{p}\right)$$

In Equation (20), $\sigma_{1},\sigma_{2},\sigma_{3},\ldots \sigma_{p}$ is the p non-zero singular values of matrix C, and $\sigma_{1}>\sigma_{2}>\cdots \sigma_{p}$. Bring (19) and (20) into (18) and obtain the solution to x as:

(21) $$x_{p}=\sum_{i=1}^{p}\sigma_{i}^{-1}v_{i}u_{i}^{T}\cdot y$$

From Equation (21), it can be seen that singular values appear in their reciprocal form in the expression of the solution. Therefore, the smaller the singular values, the greater the probability that their small changes will have a significant impact on the stability of the solution. Therefore, during the execution of the algorithm, smaller singular values have a greater impact on the stability of the filtering estimation results, which can lead to larger errors. Additionally, singular values in the matrix are arranged in descending order, with the sum of the first 10% of singular values generally accounting for 90% of the total [29]. During decomposition, most of the information is contained within the first 10% of the singular values.

Based on the above analysis, we choose to retain the larger singular values. Although discarding the smallest data points, this approach enhances the filter’s ability to resist interference from noisy data, contributing to more stable estimation results. If only the first t singular values are retained, the selection of the truncation value becomes a critical factor for the computational accuracy of this method.

### 3.3. Selection of Truncation Threshold

In the process of truncated singular-value decomposition (TSVD), the selection of truncation threshold t is particularly important. If t is too large, some small non-zero singular values may not be discarded, leading to unstable solutions and poor filter stability. Conversely, if t is too small, valuable information may be lost, resulting in poor solution accuracy. Therefore, the singular-value truncation method based on the Akaike information criterion (AIC) is employed in this context. The core principle of AIC is to balance goodness of fit with model complexity in order to avoid overfitting. The formula for AIC is given by:

(22) $$\mathrm{AIC}=2k-2\log\left(\hat{L}\right)$$

where k is the number of parameters in the model, and $\hat{L}$ is the maximum likelihood estimate. The goal of AIC is to minimize this value, thereby selecting the most suitable model. While adding parameters may improve the model’s fit, it also increases its complexity, which can lead to overfitting and reduce its generalization ability. AIC mitigates this by including the penalty term 2k, which discourages unnecessarily complex models and prevents the selection of models that overfit the data.

In the process of singular-value selection based on the AIC, the matrix A is first decomposed using SVD to obtain the singular-value vector $\sigma =\left[\sigma_{1},\sigma_{2},\ldots ,\sigma_{n}\right]$. Then, for different numbers of singular values k, the reconstruction error $\left\|A-A_{k}\right\|$ between the reconstructed matrix $A_{k}$ and the original matrix A is calculated. This error can be approximated as the negative logarithmic term in the log-likelihood function, expressed as:

(23) $$-2\log\left(\hat{L}\right)\approx {\left\|A-A_{k}\right\|}^{2}$$

Calculate the AIC value corresponding to each number of singular values k:

(24) $$\mathrm{AIC}\left(k\right)=2k+{\left\|A-A_{k}\right\|}^{2}$$

Finally, select the value of k that minimizes the AIC value as the truncation point for the singular values.

In the context of SVD used for dimensionality reduction or noise filtering, AIC can be applied to determine the optimal number of singular values to retain. Retaining too many singular values may introduce noise into the model, while retaining too few can lead to the loss of important information. By calculating the AIC value for different numbers of singular values k, the model with the lowest AIC is selected, which represents the optimal trade-off between model accuracy and complexity. This ensures not only a good fit but also reduces noise influence, thereby enhancing the system’s numerical stability.

## 4. Extended Dimension and Embedding CKF Method

Since the system noise does not satisfy the additivity condition, it is necessary to expand the dimensionality of the state variables before performing nonlinear filtering. In this section, we extend the dimensionality of the system-state variables by incorporating system noise into the state variables. The embedded cubature Kalman filter (E-CKF) algorithm is then utilized to suppress the positioning errors that arise with the increased dimensionality of the state space, thereby improving the system’s positioning accuracy.

### 4.1. Embedded Cubature Kalman Filter

The system’s state and measurement equations exhibit strong nonlinear characteristics, and the traditional CKF is no longer sufficient to resist noise interference in complex environments. In this section, we adopt the E-CKF to improve the inherent deficiencies of the spherical–radial cubature rule.

Consider infinite integrals over the real number field $R^{n}$. Using the embedded cubature criterion, we can construct a set of completely symmetrical set families R, so as to approximate the integral $I\left(f\right)$, so that $R^{\left(m,n\right)}$ is the element in r that satisfies the polynomial fitting order of 2m+1; then, Equation (28) can make the fitting accuracy of N-dimensional polynomial at most 2m+1 order. Suppose a is an n partition of the set $P^{\left(m,n\right)}$ composed of integers 0,1,⋯,m, and for i≠j, there is $P^{\left(m_{i},n_{i}\right)}\neq P^{\left(m_{j},n_{j}\right)}$, and n partitions of $P^{\left(m,n\right)}$ are arranged in descending order; then, there is Equation (25):

(25) $$P^{\left(m,n\right)}=\left\{\left(p_{1},\ldots ,p_{n}\right)\left|m\geq p_{1}\geq \cdots p_{n}\geq 0\right.,\sum_{i=1}^{n}p_{i}\leq m\right\}$$

Given a set of non-negative and different cubature point generators $u_{0},u_{1},\ldots u_{k}$, where $u_{0}=0$, Equation (26) can be defined:

(26) $$f\left[u\right]=\sum_{q\in \Pi_{p}}\sum_{s}f\left(s_{1}u_{q1},s_{2}u_{q2},\cdots ,s_{n}u_{qn}\right)$$

where $\prod_{p}$ is the set of all transformations of p and $s_{i}=\pm 1$, u meets $u=\left(u_{p1},u_{p2},\cdots ,u_{pn}\right)$. Let $\delta =\left(\delta ,\cdots ,\delta \right)$, $\delta \in R^{+}$ and the value be consistent; then, $R^{\left(m,n\right)}$ can be expressed as Equation (27):

(27) $$R^{\left(m,n\right)}\left(f\right)=\sum_{p\in P^{\left(m-1,n\right)}}W_{p}^{\left(m,n\right)}f\left[u\right]+W^{\left(m,n\right)}f\left[\delta \right]$$

where $W_{p}^{\left(m,n\right)}$ and $W^{\left(m,n\right)}$ are the corresponding weights, the value of which can completely determine the value of Equation (27). It can be seen from the theorem that for the third-order criterion, only the integration of f in the set of even-fitting polynomials $\left\{1,x_{1}^{2}\right\}$ needs to be considered, and then Equations (28) and (29) can be obtained:

(28) $$\left[\begin{array}{cc} 1 & 2^{n}\\ 0 & 2^{n}\delta^{2} \end{array}\right]\left(\begin{array}{c} W_{0}^{\left(1,n\right)}\\ W^{\left(1,n\right)} \end{array}\right)=\left(\begin{array}{c} I_{0}\\ I_{2} \end{array}\right)$$

(29) $$W_{0}^{\left(1,n\right)}=\left(1-\frac{1}{2\delta^{2}}\right)I_{0},W^{\left(1,n\right)}=\frac{1}{2^{n}\delta^{2}}I_{2}$$

By transforming the integration into a standard Gaussian distribution, Equation (30) can be derived through calculation. When the dimension of the system state variable is n, $N=2^{n}+1$ volume points can be selected to approximate the value of the integration, which can be converted into (31). The corresponding volume points and weight values are shown in (32) and (33), respectively:

(30) $$I_{N}\left(f\right)=\sum_{p\in P\left(0,n\right)}\left(1-\frac{1}{2\delta^{2}}\right)f\left[0\right]+\frac{1}{2^{n+1}\delta^{2}}f\left[\sqrt{2}\delta \right]≜\sum {\bar{\omega }}_{i}f\left(\xi_{i}\right)$$

(31) $$I_{N}\left(f\right)=\sum_{i=1}^{n}{\bar{\omega }}_{i}f\left(\xi_{i}\right)$$

(32) $$\xi_{i}=\left\{\begin{array}{l} {\left[0\right]}_{i},i=1\\ \sqrt{2}{\left[2\right]}_{i},i=2\cdots N \end{array}\right.$$

(33) $${\bar{\omega }}_{i}=\left\{\begin{array}{l} 1-\frac{1}{2\sigma^{2}},i=1\\ \frac{1}{2^{n+1}\sigma^{2}},i=2\cdots N \end{array}\right.$$

where $\left[0\right]$ is the zero vector with n-dimension, $\left[\delta \right]={\left[s_{1}\sigma^{2},\ldots ,s_{1}\sigma^{2}\right]}^{T}$ is the cubature point set, and the subscript i represents the sequence number of this basic point in the point set. Let σ=1 and $s_{1}=\pm 1$ fully arrange the elements in the n-dimension cubature point set ${\left[s_{1}\sigma^{2},\ldots ,s_{1}\sigma^{2}\right]}^{T}$. Replacing (32) and (33) with the cubature points and their weights in the cubature Kalman filter, and assuming that the initial conditions $P_{0\left|0\right.}$ and ${\hat{X}}_{0\left|0\right.}$ of the system are known, the standard E-CKF filtering framework can be constructed.

### 4.2. Extended-Dimension Embedded Cubature Kalman Filter

As the dimensionality of the state variables increases, the system noise cannot be directly separated from the state function. Therefore, before performing nonlinear filtering, it is necessary to expand the dimensionality of the system-state variables. The increase in state dimensionality, combined with the inherent nonlinearity of the system, results in a higher computational burden. During the dimensional expansion, the system’s process noise and observation noise are incorporated into the state variables, allowing the cubature points to propagate system noise information through the nonlinear functions.

Assuming that the previous state estimation value ${\hat{x}}_{k|k}$ and error covariance matrix $P_{k|k}$ in the posterior probability density function $p\left(x_{k}\right)=N\left({\hat{x}}_{k|k},P_{k|k}\right)$ at time k are known, the SVD of $P_{k|k}$ can obtain the process variable $S_{k|k}$. By adding the process noise $w_{k}$ and measurement noise $v_{k}$ of the system to the state variables, and expanding the state estimation values ${\hat{x}}_{k|k}$ and $S_{k|k}$, the process variables ${\hat{x}}_{k|k}^{a}$ and $S_{k|k}^{a}$ can be obtained, as shown in Equations (34) and (35):

(34) $$p_{k|k}=S_{k|k}S_{k|k}^{T}$$

(35) $${\hat{x}}_{k|k}^{a}={\left[\begin{array}{ccc} {\hat{X}}_{k|k}^{T} & 0_{1\times n_{w}} & 0_{1\times n_{v}} \end{array}\right]}^{T}$$

(36) $$S_{k|k}^{a}=\left(\begin{array}{ccc} S_{k|k} & 0 & 0\\ 0 & \sqrt{Q_{k}} & 0\\ 0 & 0 & \sqrt{R_{k}} \end{array}\right)$$

By substituting the above equations into the E-CKF algorithm framework presented in Section 4.1, we derive the AIC-based extended E-CKF algorithm, hereafter referred to as AEACKF. In this algorithm, two cubature transformations are performed during the prediction phase. Considering the influence of system noise, the cubature points must be regenerated before the second transformation. However, as the cubature points generated after the first transformation already incorporate noise information, we retain these points and use them directly for system computation.

### 4.3. The Method of TSVD-AEACKF

Based on the analysis above, the main steps of the algorithm proposed in this paper are as follows:

Initialize the state vector ${\hat{x}}_{0}$ and the state error covariance matrix $P_{0}$:

(37) $${\hat{x}}_{0}=E\left[x_{0}\right]$$

(38) $$P_{0}=E\left[\left(x_{0}-{\hat{x}}_{0}\right){\left(x_{0}-{\hat{x}}_{0}\right)}^{T}\right]$$

Update the time: Perform SVD on the state error covariance matrix $P_{k-1|k-1}$, where $S_{k-1}$ in Equation (39) is a diagonal matrix.

(39) $$P_{k-1|k-1}=U_{k-1|k-1}\left(\begin{array}{cc} S_{k-1|k-1} & 0\\ 0 & 0 \end{array}\right)V_{k-1|k-1}^{T}$$

Truncate the smaller non-zero singular values and retain the larger ones to obtain the new diagonal matrix (40). Then, use the process noise and measurement noise to expand the dimensionality of the state, yielding the process variables ${\hat{x}}_{k|k}^{a}$ and $S_{k|k}^{a}$ in Equations (35) and (36). The cubature points $X_{i,k-1|k-1}$ are calculated through the process variables ${\hat{x}}_{k|k}^{a}$ and $S_{k|k}^{a}$, as shown in Equation (41).

(40) $$S_{k-1}^{\prime }=diag\left(s_{1},s_{2},\ldots ,s_{n}\right),\left(j<n\right)$$

(41) $$\begin{array}{ll} X_{i,k-1|k-1} & =S_{k|k}^{a}\xi_{i}^{E}+{\hat{x}}_{k|k}^{a}\\ & =U_{k-1|k-1}\sqrt{S_{k-1|k-1}^{\prime }}\xi_{i}^{E}+{\hat{x}}_{k-1|k-1},\left(i=1,2,\ldots ,2n\right) \end{array}$$

where n is the cubature point number. According to the embedded cubature criterion in the E-CKF algorithm, replace the cubature point set and weight according to Equations (32) and (33). By propagating the state variable component and process noise component of the cubature point through the system function, the propagated cubature point can be obtained, as shown in Equation (42). Calculate the predicted state value at the time and the corresponding predicted state error covariance, as shown in Equations (43) and (44), respectively.

(42) $$X_{i,k|k-1}^{*}=f\left(X_{i,k-1|k-1},w_{k-1}\right)$$

(43) $${\hat{x}}_{k|k-1}=\frac{1}{2n}\sum_{i=1}^{2n}X_{i,k|k-1}^{*}$$

(44) $$P_{k|k-1}=\frac{1}{2n}\sum_{i=1}^{2n}X_{i,k|k-1}^{*}X_{i,k|k-1}^{*T}-{\hat{x}}_{k|k-1}{\hat{x}}_{k|k-1}^{T}+Q_{k-1}$$

Update the measurement: Perform TSVD on $P_{k|k-1}$. Preserve l larger singular values to obtain a new diagonal matrix (46):

(45) $$P_{k|k-1}=U_{k|k-1}\left[\begin{array}{cc} S_{k|k-1} & 0\\ 0 & 0 \end{array}\right]V_{k|k-1}^{T}$$

(46) $$S_{k-1}^{\prime }=diag\left(s_{1},s_{2},\ldots ,s_{l}\right),\left(l<n\right)$$

By incorporating process noise and measurement noise to expand the state dimensionality, new cubature points are computed through the process variables ${\hat{x}}_{k|k}^{a}$ and $S_{k|k}^{a}$, as shown in Equation (41). According to the cubature rule of the E-CKF, the new cubature points $\xi_{i}^{E}$ and weights ${\bar{\omega }}_{i}^{E}$ are calculated using Equations (32) and (33). The propagation of the cubature points through the measurement function is described by Equation (47), while the predicted values and residuals at time k are given by Equation (48).

The autocorrelation covariance and cross-correlation covariance are, respectively, as shown in Equations (49) and (50).

(47) $$Z_{i,k|k-1}=H_{k}X_{i,k|k-1}+v_{k}$$

(48) $${\hat{z}}_{k|k-1}=\frac{1}{2n}\sum_{i=1}^{2n}Z_{i,k|k-1},\eta_{k}=Z_{k}-{\hat{z}}_{i,k|k-1}$$

(49) $$P_{zz,k|k-1}=\frac{1}{2n}\sum_{i=1}^{2n}Z_{i,k|k-1}Z_{i,k|k-1}^{T}-{\hat{z}}_{k|k-1}z_{k|k-1}^{T}+R_{k-1}$$

(50) $$P_{xz,k|k-1}=\frac{1}{2n}\sum_{i=1}^{2n}Z_{i,k|k-1}Z_{i,k|k-1}^{T}-{\hat{z}}_{k|k-1}z_{k|k-1}^{T}$$

Update the parameters: the Kalman gain at time k, the state estimation value at time k, and the covariance estimation value of state error at time k are determined by Equations (51)–(53).

(51) $$K_{k}=P_{xz,k|k-1}P_{zz,k|k-1}^{-1}$$

(52) $${\hat{x}}_{k|k}={\hat{x}}_{k|k-1}+K_{k}\left(z_{k}-{\hat{z}}_{k|k-1}\right)$$

(53) $$P_{k|k}=P_{k|k-1}-K_{k}P_{zz,k|k}K_{k}^{T}$$

**Figure 2:**
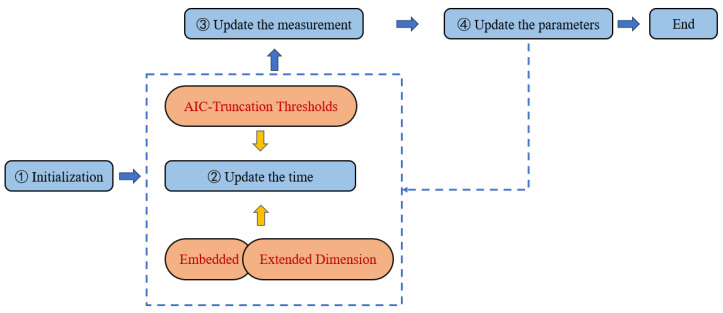
Main structure of TSVD-AEACKF algorithm.

At this point, the TSVD-AEACKF positioning process is completed. The core structure diagram of the whole algorithm is given here, as shown in Figure 2.

## 5. Experimental and Analysis

To verify the accuracy of the improved CKF algorithm, both simulation and real-world experiments were conducted. In the simulation experiments, UKF-SLAM [30], CKF-SLAM [31], and the SVD-CKF-SLAM algorithms were used as benchmarks for comparison. The effectiveness of the proposed algorithm was preliminarily validated in terms of robot localization error and runtime performance. Additionally, experiments were conducted using different observation features to analyze their impact on the state estimation of the mobile robot, as well as the accuracy of the proposed algorithm under varying noise levels. To further explore the effectiveness of the algorithm, the Car Park Dataset, an outdoor car park dataset provided by the Centre for Field Robotics Research (ACFR) at the University of Sydney [32], was used to conduct practical experiments and analyze the actual performance of the algorithm.

### 5.1. Simulation Experimental Parameters

**Figure 3:**
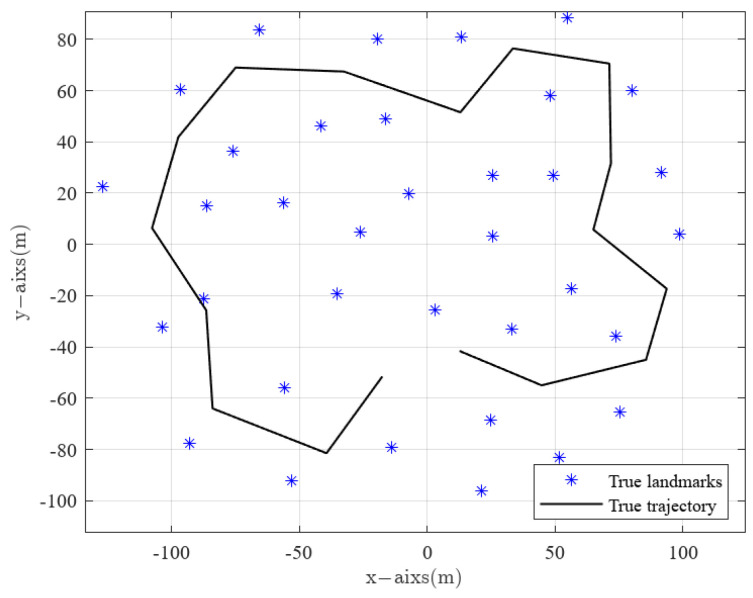
Simulation environment.

Our experiment used an Intel Core i5-10300H quad-core processor computer with 8 GB of memory. The simulation environment for SLAM is based on the MATLAB platform provided by the Sydney University Robotics Laboratory [33]. A rectangular (240 × 200 $m^{2}$) simulation environment is established. The robot’s waypoints and landmark features are manually specified. In this experiment, 17 waypoints and 35 landmark features are set. Starting from (0, 0), a mobile robot moves counterclockwise along the ideal moving path, as shown in Figure 3. The blue “*” indicates the actual landmark features, and the black “-” represents the robot’s actual trajectory.

The pose of the robot can be described by a three-dimensional state vector ${\left(x,y,\theta \right)}^{T}$, which includes its position $\left(x,y\right)$ and attitude angle θ in the global coordinate system. The attitude angle θ represents the movement direction of the robot, which is positive in the counterclockwise direction and negative in the clockwise direction, and its range is $-180^{\circ }∼180^{\circ }$. The initial state value of the robot is ${\left[0,0,0\right]}^{T}$, with a speed of v=3 m/s and an initial steering angle of G=0°; the sampling time is 0.025 s, and follows the trajectory determined by the waypoints in a counterclockwise direction, obtaining its position information based on the distance from the landmarks.

The mobile-robot motion model utilizes a front-wheel drive steering car model, with LiDAR as the onboard sensor. The simulation assumes Gaussian noise. Other parameters include a robot maximum angular velocity of g=20°/s, a velocity error of $\sigma_{v}=0.3\;m/s$, an angle error of $\sigma_{\alpha }=2{}^{\circ}$, a sensor sampling time of 0.2 s, a maximum sensing distance of 30 m, a distance observation error of $\sigma_{r}=0.1\;m$, an angular observation error of $\sigma_{\theta }=1.0{}^{\circ}$; the system noise Q and the observation noise R are as follows:

$$Q=\left[\begin{array}{cc} {\left(0.3\right)}^{2} & 0\\ 0 & {\left(\frac{3.0\pi }{180}\right)}^{2} \end{array}\right],\;R=\left[\begin{array}{cc} {\left(0.1\right)}^{2} & 0\\ 0 & {\left(\frac{1.0\pi }{180}\right)}^{2} \end{array}\right]$$

### 5.2. Analysis of Estimation Errors

In the experiments, the improved TSVD-AEACKF algorithm is compared with the actual target trajectory, and four algorithms—UKF-SLAM, CKF-SLAM, SVD-CKF-SLAM, and TSVD-AEACKF—are validated through simulation experiments under identical conditions. For each case, the final result for comparative analysis was obtained by averaging 100 independent Monte Carlo simulations.

**Figure 4:**
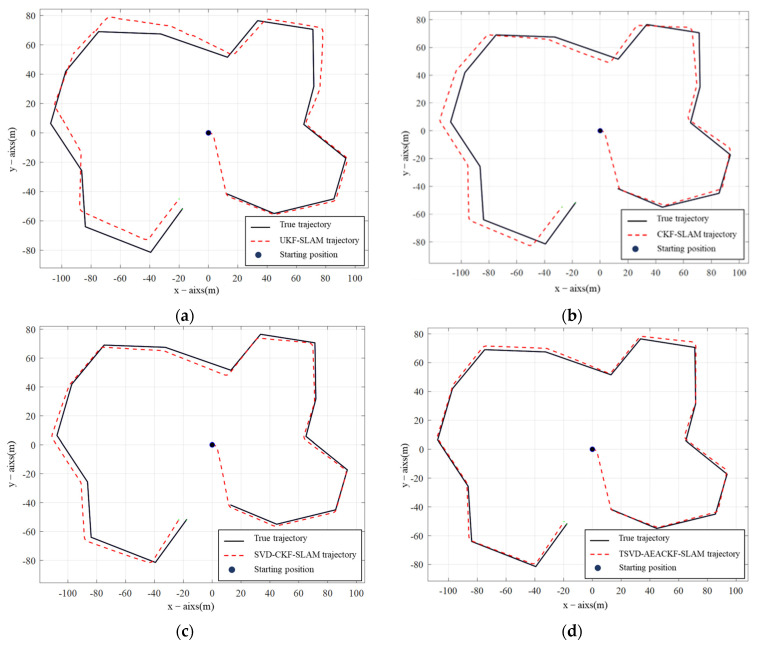
Comparison of experimental simulation results of four filtering SLAM methods. (**a**) UKF SLAM; (**b**) CKF SLAM; (**c**) SVD-CKF SLAM; (**d**) TSVD-AEACKF SLAM.

In Figure 4a–d represent the simulation results of the four algorithms: UKF-SLAM, CKF-SLAM, SVD-CKF-SLAM, and TSVD-AEACKF-SLAM. In these plots, the black trajectory represents the actual path of the mobile robot, while the red trajectory represents the estimated path produced by each algorithm. For all four algorithms, the trajectories initially converge to the ideal path; however, the estimation error grows as the environmental complexity increases over time. In Figure 4a,b, the cumulative errors of UKF-SLAM and CKF-SLAM become more pronounced with increasing time steps and updates. The filtered data for these algorithms are unstable and prone to dispersion, resulting in reduced localization accuracy. However, overall, the estimated trajectory of CKF-SLAM fits better than that of UKF-SLAM.

Figure 4c shows the estimated trajectory of SVD-CKF compared with the actual trajectory. Singular-value decomposition is introduced to enhance numerical stability based on CKF-SLAM, resulting in improved overlap with the real trajectory compared to Figure 4b. The TSVD-AEACKF-SLAM algorithm, as shown in Figure 4d, demonstrates the smallest offset between the estimated trajectory and the actual path, maintaining stability throughout the operation.

**Figure 5:**
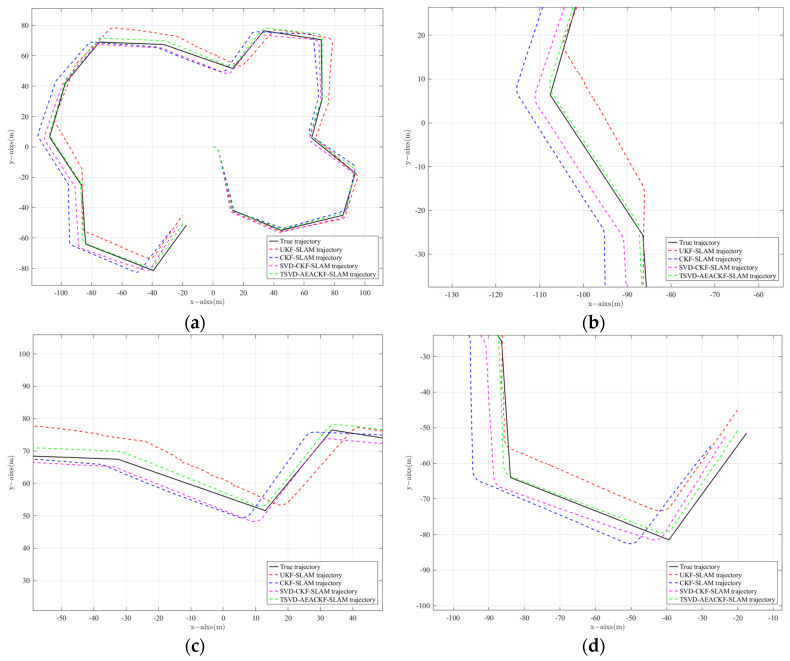
Trajectories of four SLAM algorithms. (**a**) Trajectory global; (**b**) Trajectory inflection point position 1; (**c**) Trajectory inflection point position 2; (**d**) Trajectory inflection point position 3.

Figure 5 presents the simulated trajectories of the four algorithms compared to the real trajectories, while (b) and (c) are zoomed-in views of specific regions in (a). The local scaling plots indicate that the proposed algorithm performs best at various corners, achieving the highest degree of fit with the actual trajectory of the mobile robot compared to the other three algorithms.

**Figure 6:**
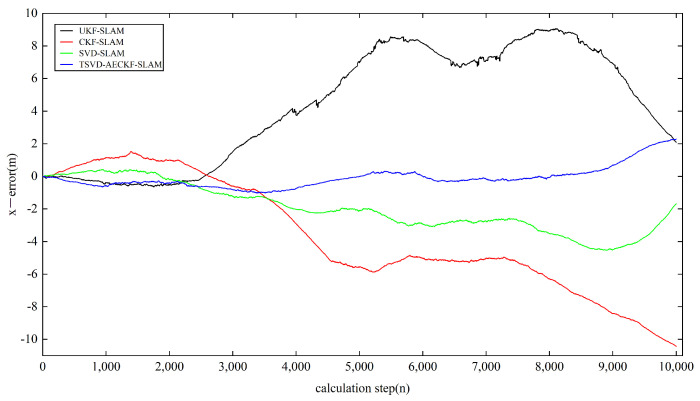
A comparison of the state localization estimation error in the x-direction of four filtering SLAM algorithms.

**Figure 7:**
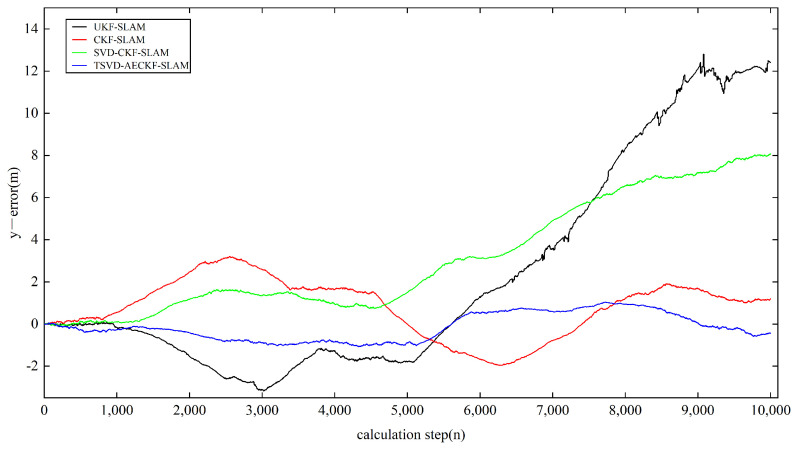
A comparison of the state localization estimation error in the y-direction of four filtering SLAM algorithms.

**Figure 8:**
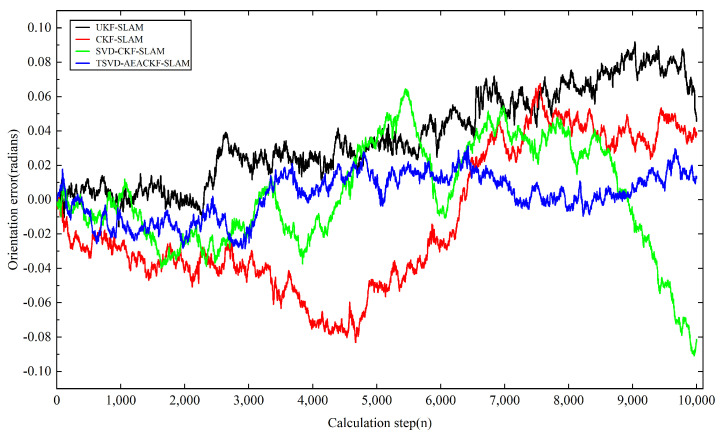
A comparison of the state localization estimation error in the orientation of four filtering SLAM algorithms.

Next, we analyzed the estimation accuracy of the four algorithms. As shown in Figure 6, Figure 7 and Figure 8, the four algorithms produce negligible path errors during the initial phase of robot operation. However, over time, the estimation errors of three algorithms—UKF-SLAM, CKF-SLAM, and SVD-CKF-SLAM—increase significantly. In the x-direction, UKF-SLAM begins to diverge at 2500 time steps, while the estimation errors of CKF-SLAM and SVD-CKF-SLAM also grow progressively from 3500 time steps. Only the TSVD-AEACKF-SLAM algorithm maintains a relatively small estimation error that remains stable throughout the robot’s movement. In the y-direction, TSVD-AEACKF-SLAM also maintains the smallest error throughout the entire process, followed by SVD-CKF-SLAM. The estimation error of UKF-SLAM grows dramatically from 5000 time steps, while the accuracy of CKF-SLAM surpasses that of SVD-CKF-SLAM after 6000 steps. In terms of attitude angle, TSVD-AEACKF-SLAM exhibits the least fluctuation throughout the process and maintains a low error state for an extended period, with values ranging between −0.03 and 0.02. This demonstrates that the TSVD-AEACKF-SLAM proposed in this paper enhances the robot’s localization accuracy and target recognition capability in the SLAM process.

### 5.3. Analysis of RMSE and Average Running Time

**Figure 9:**
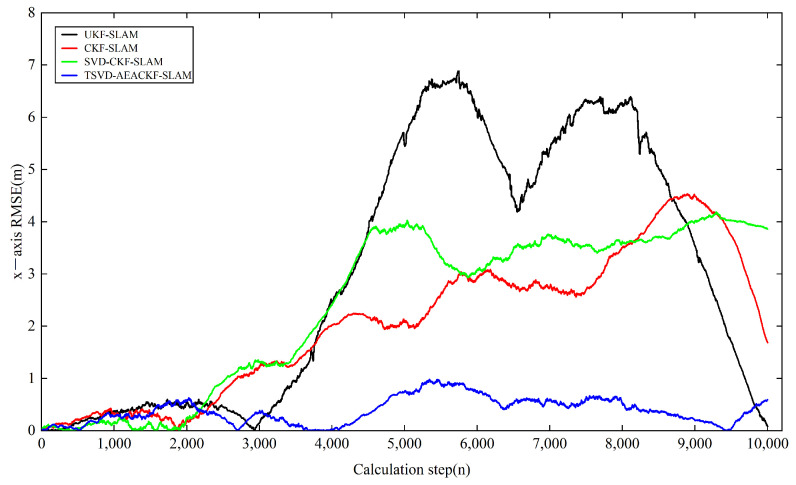
An estimation of RMSE in the x-direction.

**Figure 10:**
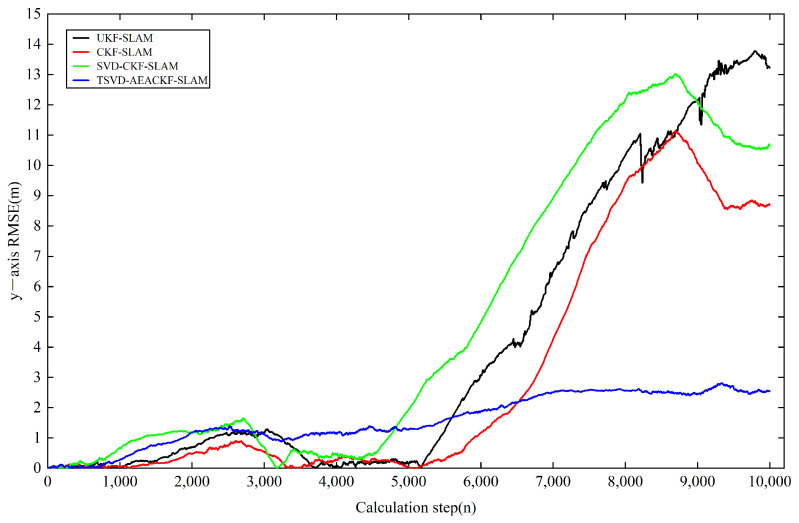
An estimation of RMSE in the y-direction.

**Figure 11:**
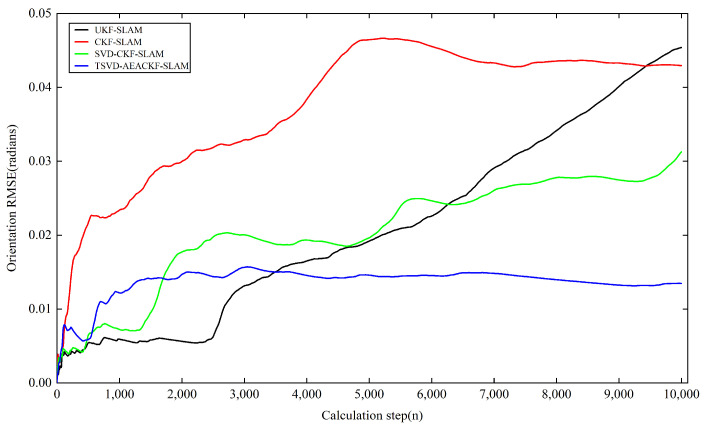
An estimation of RMSE in the orientation.

To provide a more intuitive comparison of the four algorithms, the average running time and Root Mean Square Error (RMSE) of the estimation results were calculated according to Equations (55) and (56).

(54) $$T=\frac{1}{N}\sum_{i=1}^{N}t_{i}$$

(55) $$RMSE_{x}\left(t\right)=\sqrt{\frac{1}{N}\sum_{i=1}^{N}{\left(x_{t}^{i}-{\hat{x}}_{t}^{i}\right)}^{2}}$$

(56) $$RMSE_{y}\left(t\right)=\sqrt{\frac{1}{N}\sum_{i=1}^{N}{\left(y_{t}^{i}-{\hat{y}}_{t}^{i}\right)}^{2}}$$

where T represents the average time, $t_{i}$ is the runtime at step i, and N is the number of Monte Carlo experiments conducted. $\left(x_{t},y_{t},\theta_{t}\right)$ and $\left({\hat{x}}_{t},{\hat{y}}_{t},{\hat{\theta }}_{t}\right)$ denote the true and estimated values of position and orientation at time t in the i-th experiment, respectively. The RMSE variations in the x and y directions for the four algorithms are shown in Figure 9 and Figure 10. In the x-direction, the RMSE value of the UKF-SLAM algorithm is within 10 m, while the RMSE values for CKF-SLAM and SVD-CKF-SLAM are within 5 m. The proposed TSVD-AEACKF-SLAM algorithm achieves an RMSE value of less than 1 m, indicating that its estimation accuracy surpasses that of CKF-SLAM, SVD-CKF-SLAM, and UKF-SLAM. After 3000 time steps, the RMSE value of UKF-SLAM increases rapidly due to the inclusion of a large number of landmarks, which increases the initial error of the system state. Additionally, the high degree of system nonlinearity contributes to the instability of UKF-SLAM values and an increase in non-current system errors. SVD-CKF-SLAM utilizes singular-value decomposition to update the covariance matrix, which alleviates this problem to some extent. In the y-direction, CKF-SLAM, UKF-SLAM, and SVD-CKF-SLAM algorithms all experience a sharp increase in RMSE after 6000 steps, whereas only the TSVD-AEACKF-SLAM algorithm maintains its RMSE value within 3 m, exhibiting the smallest fluctuation and highest stability. In Figure 11, the RMSE values of UKF-SLAM, CKF-SLAM, and SVD-CKF-SLAM for attitude-angle estimation increase continuously during operation. CKF-SLAM experiences a sharp increase between 0 and 500 time steps, eventually exceeding 0.04, while the RMSE value of SVD-CKF-SLAM starts to increase dramatically, followed by a similar increase in UKF-SLAM around 2500 steps. In contrast, TSVD-AEACKF-SLAM maintains numerical stability starting at 1000 steps and does not exceed a maximum RMSE of 0.015. Therefore, among the four algorithms mentioned above, TSVD-AEACKF-SLAM demonstrates the highest estimation accuracy and maintains numerical stability during mobile-robot operation.

The average running times of 100 Monte Carlo experiments performed by the four algorithms are listed in Table 1. It can be seen that UKF-SLAM has the shortest running time, while CKF-SLAM and SVD-CKF-SLAM have running times exceeding 10 s. This is primarily because both algorithms approximate nonlinear integrals using the cubic spherical–radial volume rule, which requires the computation of a set of volume points that grows exponentially with the number of state dimensions. TSVD-AEACKF-SLAM runs in 6.6689 s, which is significantly lower than SVD-CKF-SLAM without truncation, substantially improving computational efficiency while maintaining estimation accuracy.

### 5.4. Analysis of Algorithm Accuracy Under Different Noise Levels

**Figure 12:**
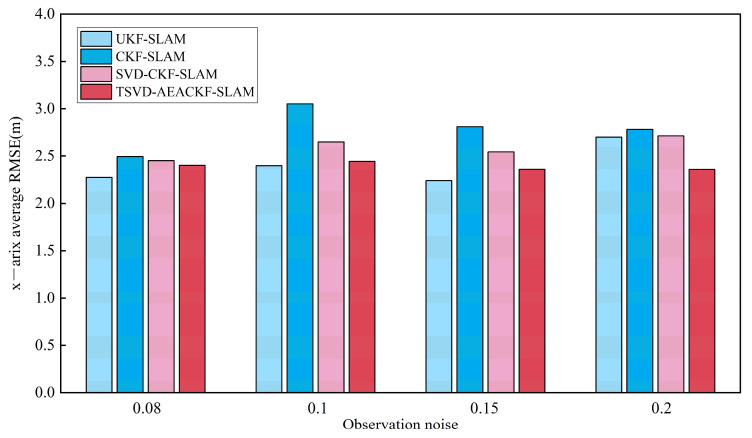
An estimation of the average RMSE for different observation noise levels in the x-direction.

**Figure 13:**
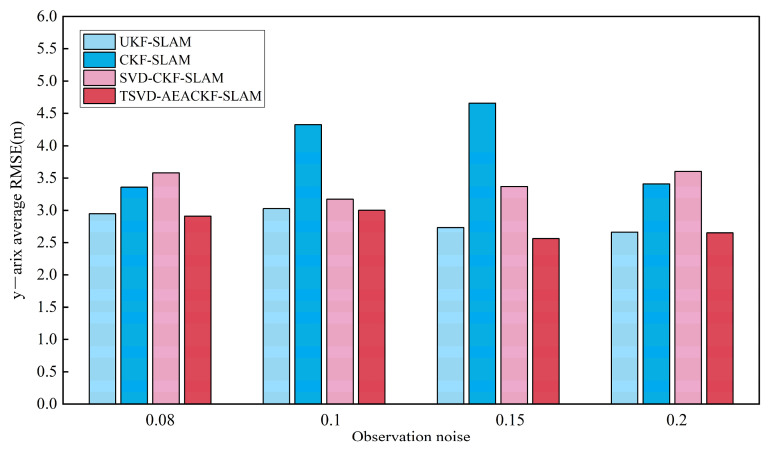
An estimation of the average RMSE for different observation noise levels in the y-direction.

**Figure 14:**
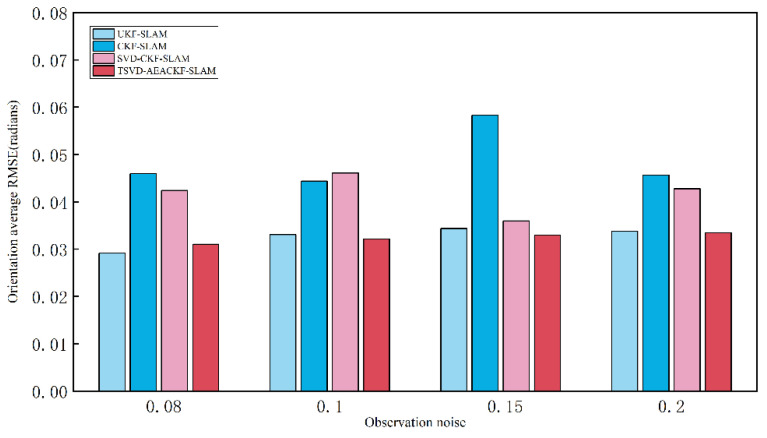
An estimation of the average RMSE for different observation noise levels in the orientation.

In applying the algorithm, different levels of system noise significantly affect its accuracy. Therefore, this section examines various system noise levels and evaluates the accuracy of the four aforementioned algorithms under the same simulation environment, keeping all other conditions constant. For each scenario, 50 Monte Carlo experiments were conducted, and the average RMSE values in the x, y, and θ directions were calculated. The observation noise $\sigma_{r}$ was set to 0.08, 0.1, 0.15, and 0.2, while the angular noise $\sigma_{\theta }$ remained constant. The average RMSE results for the x, y, and θ directions are shown in Figure 12, Figure 13 and Figure 14.

**Figure 15:**
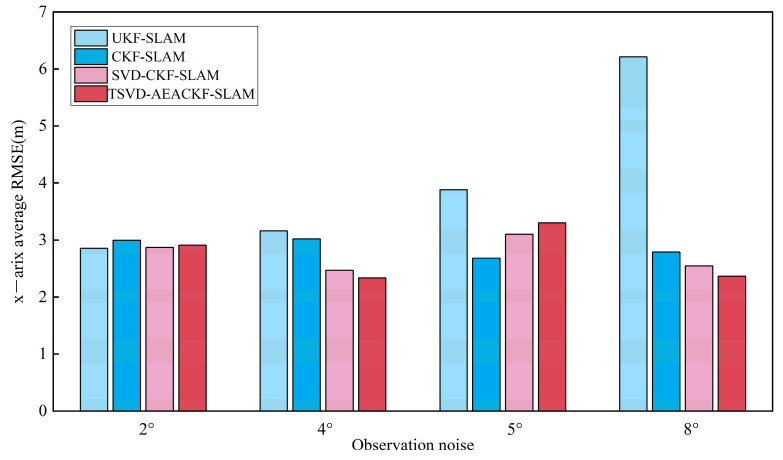
An estimation of the average RMSE for different angular noise levels in the x-direction.

**Figure 16:**
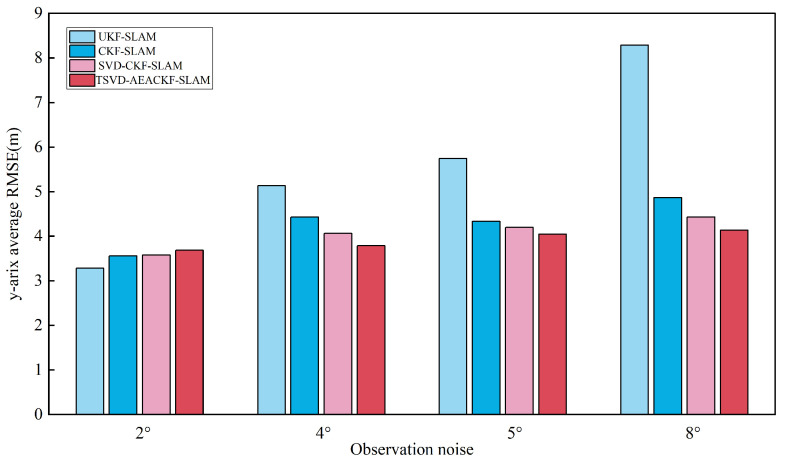
An estimation of the average RMSE for different angular noise levels in the y-direction.

**Figure 17:**
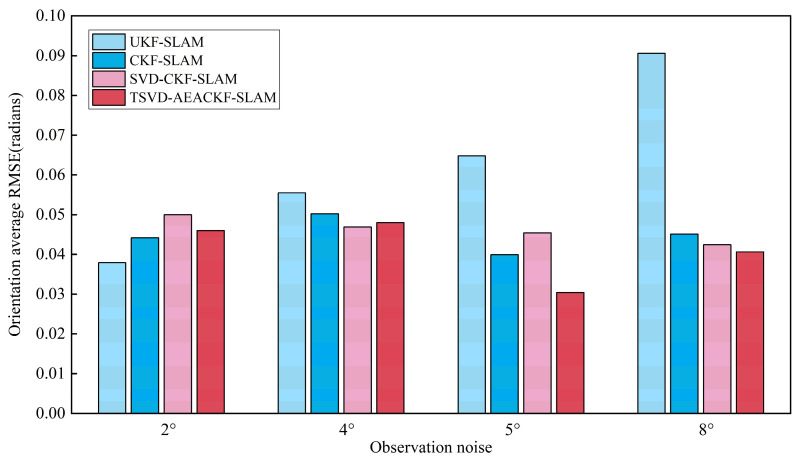
An estimation of the average RMSE for different angular noise levels in the orientation.

To investigate the effect of observation errors on the estimation accuracy of the attitude angle, the angular velocity noise $\sigma_{\theta }$ was set to 2°, 4°, 5°, and 8°, while the distance observation noise remained constant. The average RMSE results for the x, y, and θ directions are shown in Figure 15, Figure 16 and Figure 17.

The results indicate that among the four algorithms, CKF-SLAM exhibits the highest RMSE values across various distance observation noise levels. The SVD-CKF-SLAM algorithm outperforms CKF-SLAM in terms of effectiveness. Furthermore, as noise levels vary, TSVD-AEACKF-SLAM demonstrates consistent stability throughout the process, highlighting a significant advantage in accuracy. Under varying angle-related noise conditions, UKF-SLAM is the most adversely affected, with a notable increase in error values as noise levels rise. In contrast, TSVD-AEACKF-SLAM performs robustly with minimal impact, excelling in estimation accuracy compared to the other three algorithms. Overall, TSVD-AEACKF-SLAM demonstrates optimal performance across different observation-related noise levels, maintaining both accuracy and stability, which is highly beneficial for the SLAM system.

### 5.5. Validation with the Real-World Car Park Dataset

**Figure 18:**
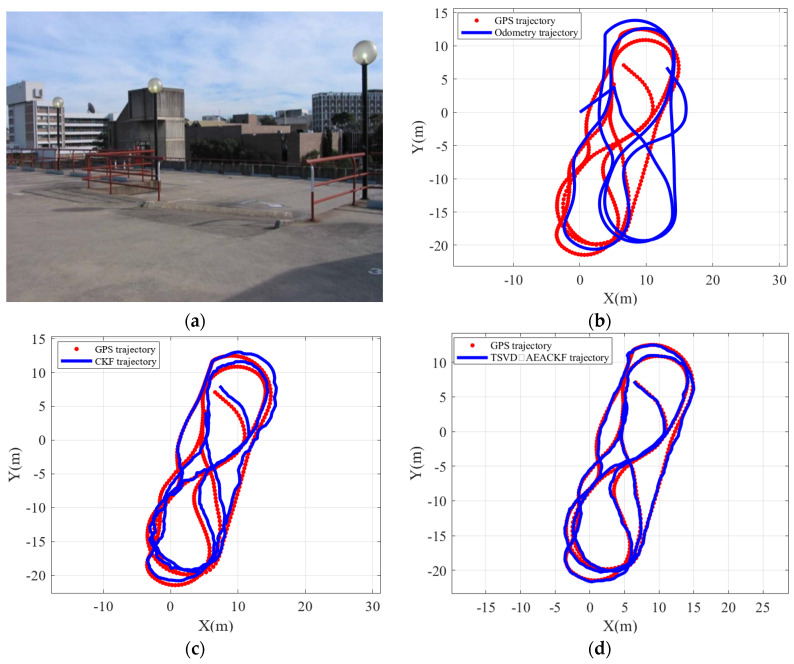
The results of SLAM in the Car Park Dataset. (**a**) Experimental environment; (**b**) Odometry trajectory; (**c**) CKF trajectory; (**d**) TSVD-AEACKF trajectory.

The Car Park Dataset is one of the commonly used datasets for studying SLAM problems, and the data collection environment is shown in Figure 18a. During the experiment, the mobile robot used for data collection was a car equipped with a SICK laser radar with a 180-degree field of view and an 80 m scanning range, as well as odometry and GPS sensors, which were used to determine the actual trajectory and evaluate the effectiveness of the SLAM algorithm. Figure 18b shows the GPS trajectory of the robot and the odometry-based dead-reckoning trajectory. As seen in the figure, due to odometry measurement noise and inaccuracies in the motion model, the dead-reckoning trajectory diverges significantly, leading to severe deviation from the GPS trajectory. Based on this dataset, this section applies the TSVD-AEACKF algorithm and the standard CKF SLAM algorithm in real-world experiments, comparing them with GPS trajectory data to validate the performance of the improved algorithm in a real environment. The results are shown in Figure 18c,d.

**Figure 19:**
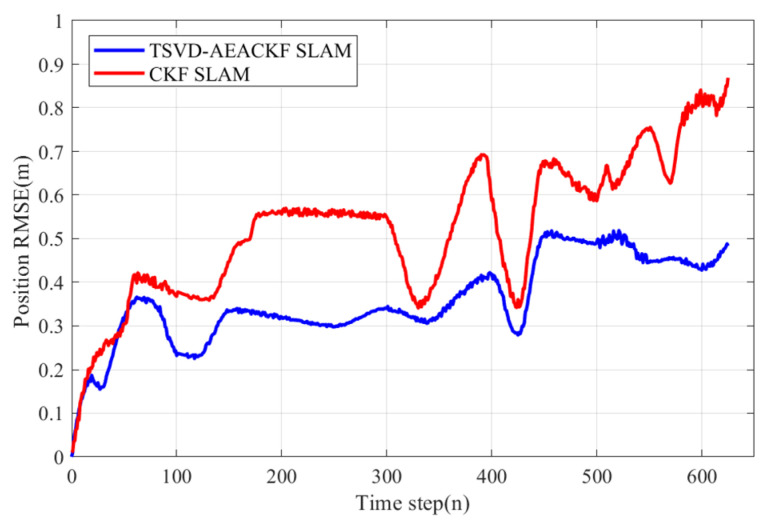
A comparison of vehicle-position RMSE in the Car Park Dataset.

The results indicate that, compared to the standard CKF SLAM algorithm, the proposed algorithm yields a trajectory that more closely matches the GPS trajectory, demonstrating reduced pose estimation errors and superior performance. A quantitative comparison of the RMSE of the robot’s position is presented in Figure 19, using GPS data points as the actual robot positions. The RMSE values for the estimated results obtained by the two algorithms are calculated, with the overall RMSE for the CKF algorithm being 0.649 m, and the improved algorithm achieving an RMSE of 0.421 m, representing an improvement of 35.13%. This indicates that the proposed algorithm effectively enhances the accuracy of robot position estimation. From the perspective of real-world performance, the improvements made by the algorithm have practical value.

## 6. Conclusions

This paper proposes a scalable embedded CKF algorithm based on TSVD-AECKF to address the issue of non-positive definiteness in the state covariance matrix during state estimation. SVD is used in place of Cholesky decomposition to optimize the iterative process. Singular values are truncated to mitigate their effect on system stability, and a selection method based on the AIC truncation threshold is provided. Additionally, to address the increasing trajectory estimation bias due to rising system dimensionality, system noise is incorporated into the state variables, and dimensionality is expanded. Finally, simulation and comparison experiments show that the TSVD-AEACKF algorithm-based mobile robot achieves the highest consistency with the real path, superior SLAM positioning accuracy, optimal stability throughout the process, and a 55.54% improvement in time efficiency compared to similar SVD-CKF algorithms, along with a 35.13% improvement in position estimation accuracy compared to the standard CKF algorithm. Future work includes further analysis of real-world conditions and the application of the proposed method to multi-sensor fusion for navigation and mapping.

## Figures and Tables

**Table 1:** Running time and RMSE of different algorithms.

Algorithm	Time (s)
UKF-SLAM	2.9839
CKF-SLAM	13.0582
SVD-CKD-SLAM	15.0007
TSVD-AEACKF-SLAM	6.6689

## Data Availability

The simulation environment of the Sydney University Robotics Laboratory is obtained from: http://www.acfr.usyd.edu.au/homepages/academic/tbailey/software.html (accessed on 6 June 2024).
